# IgE-mediated cocoa allergy: a pediatric case report and review of the literature

**DOI:** 10.3389/fped.2026.1763504

**Published:** 2026-04-13

**Authors:** Lucrezia Sarti, Susanna Morelli, Mattia Giovannini, Simona Barni, Giulia Liccioli, Leonardo Tomei, Benedetta Pessina, Gabriele Simonetti, Antonella Valenza, Claudia Valleriani, Alberto Martelli, Francesca Mori

**Affiliations:** 1 Allergy Unit, Meyer Children’s Hospital IRCCS, Florence, Italy; 2 Department of Health Sciences, University of Florence, Florence, Italy; 3 Pediatric Unit, Rho and Garbagnate Milanese Hospital, ASST-Rhodense, Milan, Italy; 4 Department of Experimental and Clinical Medicine, University of Florence, Florence, Italy; 5 Immunology Laboratory, Meyer Children’s Hospital IRCCS, Florence, Italy; 6 School, Family, and Association Task Force, Italian Society of Pediatric Allergy and Immunology, Milan, Italy

**Keywords:** anaphylaxis, case report, children, chocolate allergy, cocoa allergy, food allergy

## Abstract

**Background:**

Suspected allergic reactions to cocoa are frequently reported by patients, yet most of these cases are caused by sensitization to other more common allergens contained in chocolate products, such as milk, peanuts or tree nuts. True immunoglobulin E (IgE)-mediated cocoa allergy is rare, with only a few confirmed cases published to date.

**Case presentation:**

We describe the case of a 2-year-old female with a history of allergic rhinitis and anaphylaxis to tree nuts, who experienced recurrent episodes of perioral erythema and angioedema following chocolate ingestion. Skin prick testing, prick-by-prick testing with cocoa products, and serum specific IgE confirmed sensitization to cocoa. An oral food challenge with a dark chocolate bar was performed under controlled hospital conditions and resulted positive. The patient developed immediate multisystemic clinical manifestations including cough, wheezing, pruritus, perioral erythema, and urticaria, consistent with anaphylaxis. As the reaction occurred in a controlled hospital setting and the symptoms resolved rapidly with oral antihistamines, corticosteroids, and inhaled salbutamol, intramuscular epinephrine was not administered. The patient was discharged in good condition with a strict dietary avoidance of cocoa.

**Conclusion:**

This case describes a rare but definite diagnosis of IgE-mediated cocoa allergy confirmed by oral food challenge. Diagnostic assessments should carefully exclude hidden allergens and consider alternative mechanisms, including contamination of cocoa products during processing or manufacturing. Clinicians should be aware that, although uncommon, cocoa allergies can cause anaphylaxis, and a comprehensive diagnostic workup, including oral challenge, is essential to guide correct management and patient counselling.

## 1 Introduction

Suspected allergic reactions to cocoa are quite commonly reported by patients, and they are a frequent reason for allergy consultation (1). In most cases, such reactions can be attributed to contamination with common allergens that are often mixed with chocolate and cocoa products, such as peanuts, milk or tree nuts (2).

Although true immunoglobulin E (IgE)-mediated allergy to cocoa is rare and has been described only in a few cases in the literature, an accurate allergy work-up is essential for its diagnosis. Additionally, it is crucial to distinguish true IgE-mediated cocoa allergy from other conditions occasionally associated with chocolate ingestion, such as contaminations from other hidden allergens (3), pseudoallergic reactions or nickel-related systemic contact dermatitis (4).

We describe the case of a pediatric patient with a confirmed IgE mediated cocoa allergy, documented through positive skin prick test (SPT), serum specific IgE (sIgE) and oral food challenge (OFC).

To the best of our knowledge, this is one of the few pediatric cases of confirmed IgE-mediated cocoa allergy.

## 2 Case report

A 2-year-old female was referred to our Allergy Unit with a history of allergic rhinitis and food allergy with several systemic reactions, such as anaphylaxis to hazelnuts and walnuts. Considering the history of severe reactions to tree nuts, a desensitization program for hazelnut was previously attempted but failed because of poor adherence. During follow-up, she reported two episodes of perioral erythema and angioedema of the lips after consuming milk chocolate [reported allergens: Milk Powder, Cocoa Butter, Cocoa Mass, Lecithin (soy/sunflower), natural identical vanillin], without the possibility of the presence or traces of tree nuts. She followed an elimination diet for walnuts, hazelnuts and cocoa/chocolate, but routinely consumed milk, soy and other possible allergens without problems. Notably, prior to these episodes, she had never consumed chocolate and, therefore, had never experienced any adverse reactions to chocolate products.

**Figure 1:**
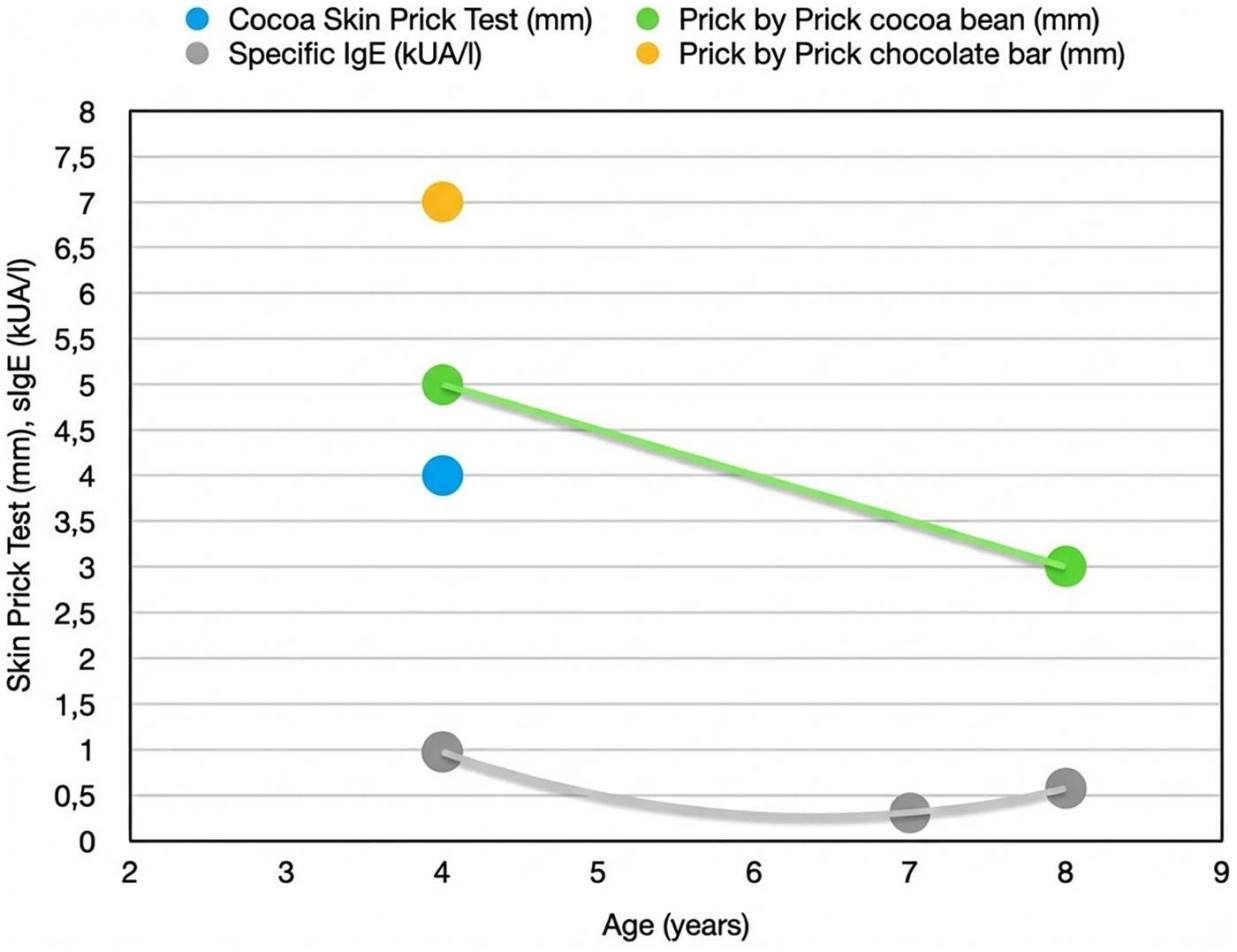
Skin prick test (SPT), prick by prick (PbP) test and serum specific IgE (sIgE) results in our patient over time.

At follow-up, the SPT with cocoa extract (Lofarma, Milan, Italy) showed a 4 mm wheal, and prick by prick (PbP) tests resulted positive with a 5 mm wheal for cocoa beans and a 7 mm wheal for chocolate bars. The positive and negative controls for SPTs were obtained using histamine (10 mg/ml; Lofarma, Milan, Italy) Additionally, sIgE was 0.97 kUA/L using the ImmunoCAP® system (Thermo Fisher Scientific, Uppsala, Sweden) (Figure 1). Therefore, the patient was advised to exclude cocoa from her diet.

**Figure 2:**
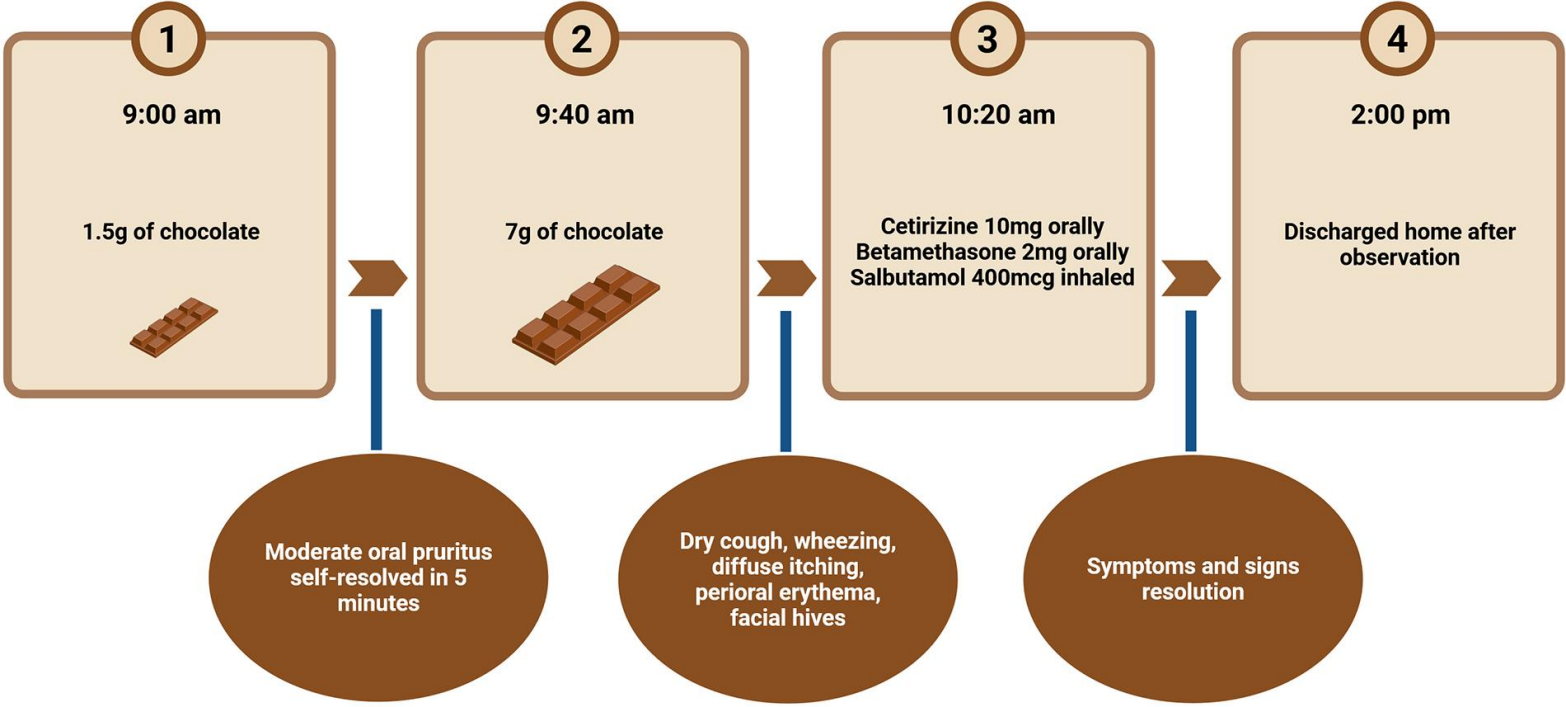
Protocol of oral food challenge with cocoa used for our patient. Created with BioRender.com.

Considering the clinical history, skin test results, and the patient's and family's willingness to participate, informed consent was obtained, and a cocoa OFC was scheduled. During the OFC, the patient consumed a 70% dark chocolate bar with mention of possible traces of almonds (always tolerated by the patient) without alerting for possible presence, even traces of other tree nuts manufactured in an allergy-conscious facility with strict cross-contamination precautions. The OFC was carried out with the administration of 1.5 g and 7 g of chocolate (0.1 g and 0.5 g of protein), every 30 min (cumulative dose of 8.5 g of chocolate and 0.6 g of protein) and, after 40 min from the last dose intake the patient started to show respiratory involvement with dry cough, wheezing, and skin manifestations with diffuse itching, perioral erythema and facial hives, fulfilling the diagnostic criteria for anaphylaxis (5). Although current guidelines recommend intramuscular epinephrine as the first-line treatment for anaphylaxis, epinephrine was not administered in this case because the reaction occurred in a strictly controlled hospital setting under continuous medical supervision, with immediate availability of emergency treatment. The clinical manifestations rapidly improved with oral antihistamines, oral corticosteroids, and inhaled salbutamol. After a period of observation, she was discharged in good general condition without any clinical manifestations. The OFC test result was positive (Figure 2), and the patient was advised to strictly avoid cocoa in the future. She is currently continuing a cocoa-free diet and has been free from accidental exposure reactions.

## 3 Discussion and review of the literature

### 3.1 Cocoa production

Cocoa is obtained from the seeds of the fruit of the cocoa tree (*Theobroma cacao*), which is dried and fully fermented (6). Cacao trees belong to the *Malvaceae* family, which includes species such as durian fruit (*Durio*) and cotton (*Gossypium*). Cocoa beans are roasted, shelled, and fermented to obtain cocoa liquor, which is reported as “percent cacao” on food packaging. Cocoa powder is produced by partially removing the cocoa butter from the processed liquor (6). Chocolate is prepared by combining different parts of cocoa liquor, cocoa butter, and sugar, with the eventual addition of condensed or powdered milk to obtain milk chocolate (6).

### 3.2 Cocoa in the human diet and health

Cocoa represents a rich source of vitamins, minerals, and polyphenols, which are present in up to 50mg/g of cocoa powder, more than what is present in most foods (6). Among polyphenols, the especially high content of flavonoids in cocoa determines the upregulation of antioxidant defenses and has a favorable impact on the vascular endothelium, enhancing nitric oxide production, with a possible decrease in cardiovascular diseases (6, 7). Antioxidant effects also appear to reduce insulin resistance and the risk of diabetes (6). Furthermore, dark chocolate appeared to exert prebiotic effects by restructuring the diversity and abundance of intestinal bacteria (8). Few studies on animal models have investigated the impact of cocoa intake on the immune status, suggesting a possible role in the prevention of some immune-mediated diseases. Specifically, a study on ovalbumin (OVA)-sensitized rats showed lower anti-OVA IgE synthesis and lower total serum IgE, with reduced lymph node TNF-α and IL-10 production. These findings were attributed to the possible immunomodulatory role of the high concentration of flavonoids in cocoa (9). Accordingly, a study conducted on young adults showed a lower percentage of allergic people in moderate and high cocoa consumers (> 7 g/day), and fewer allergic manifestations in moderate cocoa consumers (10). Thus, a positive effect of cocoa intake on allergies might be hypothesized (10).

### 3.3 IgE-mediated cocoa allergy

Although IgE-mediated food allergies to cocoa are possible, limited data are currently available. Self-reported cocoa allergy is quite common, and population studies have reported a prevalence of 0.5-0.7% in selected populations in Central America (1). Although self-reported allergies are variably prevalent (11), such data are not always accurate, and most of these cases are often not allergic or are, sometimes, determined by cross-contamination, as previously discussed.

As we know, these self-diagnosed prevalences are usually overestimated, although it is impossible to provide more precise data, assuming that, depending on consumption and different geographical areas, prevalences may vary. One study found that the prevalence of cocoa allergy was as high as 1%; however, this was a study of adult patients in Saudi Arabia (12).

Although no cocoa allergen has yet been characterized, a 2S cocoa seed albumin storage polypeptide presents high sequence similarity (>52%) to other allergenic plant 2S albumins, such as those found in Brazilian nut (13). A minor allergen from walnut (vicilin family) shows approximately 90% sequence homology with the vicilin precursor from cocoa (vicilin B) (14).

Sensitization to cocoa mainly occurs via ingestion; however, it has also been reported to occur via inhalation owing to occupational exposure. Reports on chocolate confectionery workers have shown a high level of cocoa sensitization (up to 31%) with an increased prevalence of asthma and dyspnea in the subgroup of sensitized confectionery workers (15).

Data on challenge-proven type I hypersensitivity to cocoa are still limited: other than our work only a recent case series reported three pediatric patients with suspected allergy to chocolate/cocoa (16). The SPTs were persistently positive, whereas sIgE in two cases appeared to markedly decrease over time; nevertheless, the OFC resulted positive, and consistent with anaphylaxis, in all the reported cases (16). Additionally, a recent case report described a 24-year-old woman with previous anaphylaxis to walnuts and almonds who developed anaphylaxis within minutes after ingesting dark chocolate (17). SPT and PbP testing confirmed sensitization to cocoa, and *in vitro* analyses identified IgE-binding proteins in cocoa extracts; however, no OFC was performed in this case (17).

We present a rare case of cocoa type I hypersensitivity in a patient with tree nut allergy confirmed by a positive OFC resulting in anaphylaxis according to the World Allergy Organization anaphylaxis guidance (18).

### 3.4 Allergic reaction to hidden allergens in chocolate products: cross-contamination

Chocolate is a complex food matrix and may contain undeclared allergens, particularly milk, hazelnuts, almonds, peanuts, or soy. These hidden allergens are often responsible for allergic reactions following chocolate ingestion (2). Therefore, when evaluating a possible allergic reaction to cocoa, it is fundamental to exclude the potential involvement of other declared or undeclared allergens in the product. Highly sensitive and rapid methods such as liquid chromatography coupled with tandem mass spectrometry have been developed to detect the most common potential contaminants (19, 20). In the United States, in 75% of chocolate bars with milk advisory statement milk concentrations were found above the limit of quantitation [2.5 microg/g (ppm)], mostly >1,000 microg/g (21). Furthermore, chocolate bar products with “traces of milk” alert contained 3 to 6,700 microg/g of milk (21). The analysis of chocolate products for peanuts showed that 8% of those with an advisory statement contained peanuts (highest quantitation of 550 microg/g) (21); while all products with “peanut-free” statement were negative in the absence of such labels, 17% of products tested positive (9–170 microg/g) (21). This concern was confirmed by a more recent study analyzing recalls associated with food allergens in Food and Drug Administration (FDA)-regulated foods: 120 recalls in total, of which 42 were for milk, 25 for peanuts, 20 for tree nuts, 7 for soy, 3 for wheat, and 1 for egg, recorded from 2013 to 2019 (22). A recent study in Canada analyzed the risk of allergic reactions in milk-allergic individuals consuming products with precautionary allergen labelling (PAL) for milk and found that the estimated risk was higher for dark chocolate (16%) than for baked goods (3.8%) or cookies (0.6%) (23).

For vegan children or teenagers, who also have an allergy to cow's milk proteins, there may be a risk of an allergic reaction, even more severe, such as anaphylaxis (24).

The presence of undeclared allergens in food labeling was also studied using the Rapid Alert System for Food and Feed notifications in the European Union, highlighting a considerable percentage of notification (for milk, peanuts and tree nuts) in cocoa and cocoa preparation (25). In our case, the potential role of hidden or alternative allergens was carefully evaluated. The chocolate product used for the OFC carried a precautionary statement regarding possible traces of almond only, a food previously tolerated by the patient, and no labelling for other tree nuts. Although independent laboratory verification of protein content was not performed, the exclusion of alternative allergens was supported by the patient's documented clinical tolerance, the absence of sensitization to the declared ingredients, and the concordant results of skin testing, sIgE, and OFC indicating cocoa as the triggering allergen.

### 3.5 Pseudoallergic reactions

Pseudoallergic reactions are non-IgE-mediated responses resulting from non-specific mast cell activation or impaired degradation of biogenic amines (26). Although chocolate is not among the foods with the highest histamine content, it may act as a histamine liberator or promote endogenous histamine release, particularly in individuals with reduced activity of diamine oxidase (DAO), the main enzyme responsible for dietary histamine metabolism (26, 27). In these patients, histamine accumulation may exceed the individual degradation threshold and trigger clinical manifestations that mimic classical allergies, including flushing, pruritus, urticaria, headache, hypotension, abdominal pain or diarrhea. Similar reactions have been described following the ingestion of other histamine-releasing or histamine-rich foods such as tomatoes, aged cheese, fish, and alcoholic beverages. Individual tolerance varies substantially and chocolate-induced clinical manifestations typically depend on the overall histamine load rather than on cocoa-specific sensitization.

These reactions are classified as pseudoallergic because they do not involve IgE-mediated immune mechanisms and SPT or sIgE assays are negative. Diagnosis relies on clinical history, exclusion of true food allergies, improvement after a low-histamine diet, and, when necessary, oral histamine provocation (26, 27). Management includes the avoidance of histamine-rich or histamine-releasing foods and the use of antihistamines in patients with clinical manifestations.

In our case, the clinical manifestations following cocoa ingestion and positive test results supported an IgE-mediated mechanism rather than histamine intolerance.

### 3.6 Nickel-related manifestations

Nickel is the most common cause of contact allergy in the general population and is typically associated with type IV hypersensitivity reactions, such as allergic contact dermatitis (4, 28). Patch testing represents the diagnostic gold standard (4). While most sensitizations occur through skin exposure (e.g., jewellery, cosmetics, and metal tools), a minority of nickel-sensitized individuals may also develop systemic clinical manifestations after the ingestion of nickel-rich foods (4).

Nickel is widely present in food items, especially in cereals (whole wheat, rye, oats and soy), cocoa, tea, gelatin, baking powder, soy, red kidney beans, legumes (green beans, peas and peanuts), tree nuts, seeds, vegetables (spinach), strong licorice, dried fruits and also in nickel-containing foods and drinks (tuna, maize and beer) (29).

Cocoa and dark chocolate are known to contain relatively high amounts of nickel, partly because of natural uptake from the soil and partly because of prolonged contact with stainless-steel equipment during processing. Systemic Nickel Allergy Syndrome (SNAS) is an uncommon condition characterized by contact dermatitis associated with systemic manifestations such as delayed-onset eczema, gastrointestinal symptoms (abdominal pain, diarrhea and nausea), and occasionally urticaria or angioedema following the ingestion of nickel-containing foods (30, 31). These clinical manifestations seem to correlate with nickel dose intake, usually arising hours to days after exposure and are not immediate, in contrast to IgE-mediated reactions. Diagnosis of SNAS relies on clinical history, a positive patch test for nickel, and improvement after a low-nickel diet, and can be confirmed by an oral provocation with nickel sulphate. Although chocolate-related cases of SNAS are possible, there are currently no such reports in the literature. A case series described four cases of nickel-sensitized pediatric patients with acute generalized flares of dermatitis 48–96 h after Easter, supposedly related to a previous chocolate binge during Easter egg hunting (32). In this case, complete diagnostic tests to exclude cocoa IgE-mediated allergy were not performed, probably because the patient's history was not consistent with a type I hypersensitivity reaction. Another article reported the case of a teenager with delayed systemic contact dermatitis with skin lesions exacerbation, supposedly related to cocoa consumption; sIgE for cocoa were negative (33).

In our case, the rapid onset of clinical manifestations after chocolate ingestion, the absence of previous contact dermatitis and the positive OFC supported an IgE-mediated cocoa allergy rather than a nickel-induced reaction.

## 4 Conclusion

In conclusion, although suspected reactions to cocoa and chocolate are frequently reported, only a minority are caused by true cocoa allergies. Most cases are instead related to hidden allergens, cross-contamination with milk or tree nuts, or non-IgE-mediated mechanisms. However, our case confirms that real IgE-mediated cocoa allergy does exist, although it remains rare.

**Figure 3:**
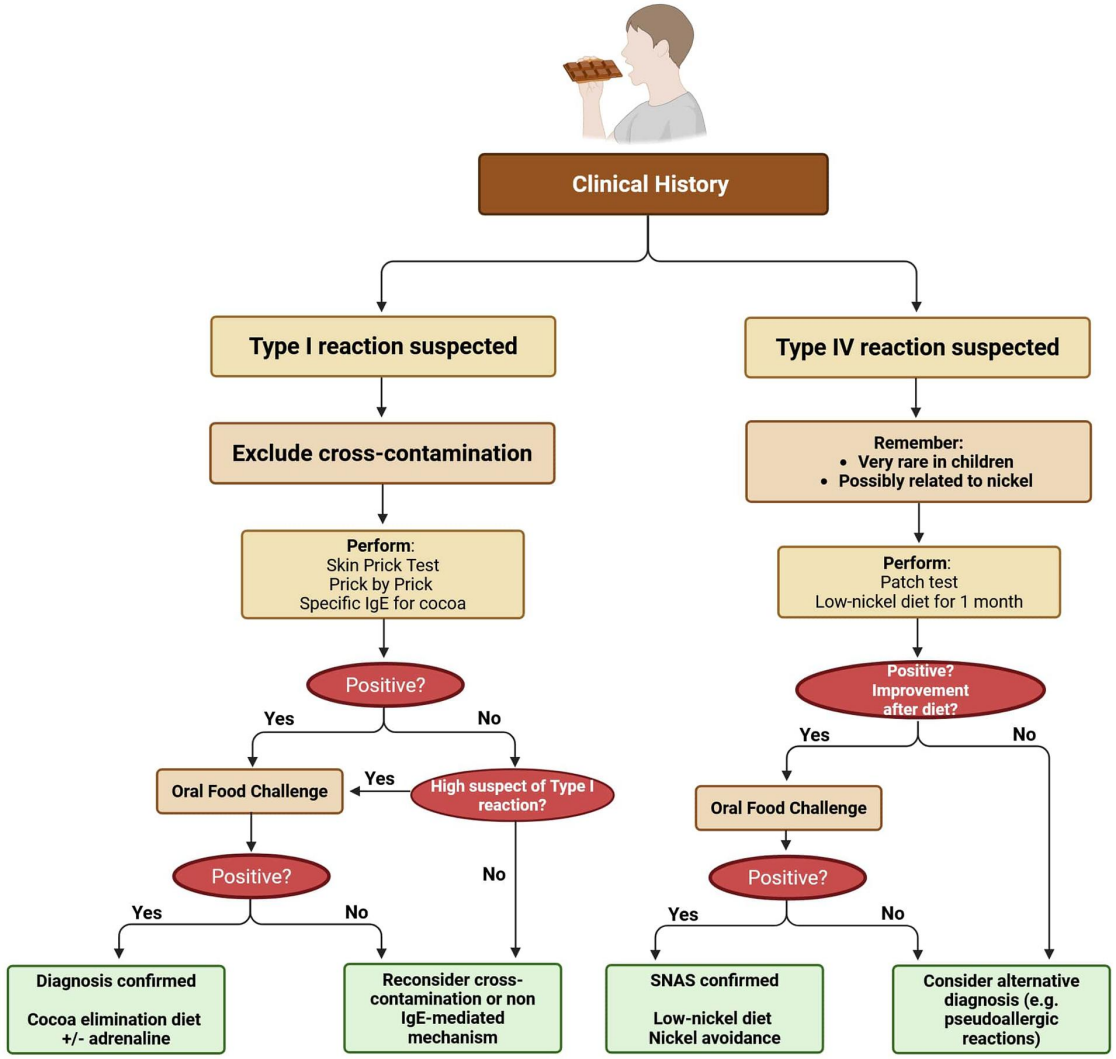
Diagnostic flowchart for suspected reactions to cocoa or chocolate. Created with BioRender.com.

Given the diagnostic complexity, we propose a diagnostic flowchart to guide clinicians in evaluating suspected reactions to cocoa or chocolate products (Figure 3). In the diagnostic process, it is crucial to rule out possible contaminations from other hidden allergens, which are not always indicated on the label, and to evaluate differential diagnoses such as pseudoallergic reactions or SNAS, especially when clinical manifestations are atypical or delayed. It is also essential to complete the assessment through a comprehensive allergy diagnostic workup, including skin tests, sIgE, and, when appropriate, an OFC to confirm the diagnosis (34).

Furthermore, given the high possibility of contamination (with milk, peanuts and tree nuts), it is crucial to advise consumers with food allergies to be cautious when consuming chocolate products, particularly those with advisory label statements. Our case highlights that appropriate dietary elimination advice, careful counselling regarding the risk of cross-contamination in chocolate products, and the prescription and correct use of epinephrine auto-injectors are essential components of patient and caregiver education. We believe that the present report may raise awareness of the potential severity of true cocoa allergies, supporting the need for careful diagnostic evaluation and cautious dietary management in tree-nut sensitized patients.

## Data Availability

The original contributions presented in the study are included in the article/Supplementary Material, further inquiries can be directed to the corresponding author/s.
